# Multi-gas pollutant detection based on sparrow search algorithm optimized ALSTM-FCN

**DOI:** 10.1371/journal.pone.0310101

**Published:** 2024-09-13

**Authors:** Xueying Kou, Xingchi Luo, Wei Chu, Yong Zhang, Yunqing Liu

**Affiliations:** School of Electronic Information Engineering, Changchun University of Science and Technology, Changchun, China; Al-Nahrain University, IRAQ

## Abstract

It is critical to identify and detect hazardous, flammable, explosive, and poisonous gases in the realms of industrial production and medical diagnostics. To detect and categorize a range of common hazardous gasses, we propose an attention-based Long Short term memory Full Convolutional network (ALSTM-FCN) in this paper. We adjust the network parameters of ALSTM-FCN using the Sparrow search algorithm (SSA) based on this, by comparison, SSA outperforms Particle Swarm Optimization (PSO) Algorithm, Genetic Algorithm (GA), Gray Wolf Optimization (GWO) Algorithm, Cuckoo Search (CS) Algorithm and other traditional optimization algorithms. We evaluate the model using University of California-Irvine (UCI) datasets and compare it with LSTM and FCN. The findings indicate that the ALSTM-FCN hybrid model has a better reliability test accuracy of 99.461% than both LSTM (89.471%) and FCN (96.083%). Furthermore, AdaBoost, logistic regression (LR), extra tree (ET), decision tree (DT), random forest (RF), K-nearest neighbor (KNN) and other models were trained. The suggested approach outperforms the conventional machine learning model in terms of gas categorization accuracy, according to experimental data. The findings indicate a potential for a broad range of polluting gas detection using the suggested ALSTM-FCN model, which is based on SSA optimization.

## Introduction

With the rapid development of industry and the improvement of people’s living standards, polluting gases from farms, vehicles, factories and other sources have a negative impact on air quality, which in turn contributes to climate deterioration [1]. There are many harmful gases, such as ammonia, ethylene, benzene, etc., which may directly cause harm to people’s health. The WHO states that air pollution is the leading cause of death for individuals with cardiovascular disease, including stroke, cancer, and chronic respiratory conditions, and that it may exacerbate asthma, particularly in children [2], that more than 32% of deaths are caused by air pollution, and that approximately 6.7 million people die prematurely each year due to air pollution, adversely affecting overall well-being, the climate and the economy [3]. Thus, it is crucial to accurately identify these gases in order to raise living standards and guarantee the safety of industrial output.

Common gas classification methods include chromatography, mass spectrometry and so on, in contrast, electronic nose (E-Nose) are available due to their low price, portability and ease of manufacture, and has been widely used in medical diagnosis, air pollutant detection [4], process control application, and urban greenhouse gas emission and other fields [5–8]. A sensor array system made up of chemical sensors is called an electronic nose. The system must also use an algorithm model to analyze the data in order to reliably extract and categorize crucial information. Choosing an appropriate categorization strategy is essential to enhancing the performance of an electronic nose. However, one algorithmic model cannot be applied to all electronic nose systems. Because of this, researchers will choose several categorization models based on various issues. Zhang et al. used SVM model to classify CO, CH_4_ and CH_2_O gases. The authors extracted the time features of sensor data as the input of SVM, and the accuracy was 98.73%, 100% and 97.46%, respectively [9]. Shakya et al. successfully used the RF model to distinguish three types of beer with 98.6% accuracy, improving the recognition and classification ability of the gas sensor [10]. Yan et al. used the LeNet5 model to effectively classify rice gas information [11]. When selecting the classification model, the amount and type of data of the sensor array should be considered. The dataset selected in this study is large, and the sensor data are usually time series at high latitude, so it is more appropriate to choose neural network for gas classification. Therefore, this study chooses to use the ALSTM-FCN model to classify the sensor array data. The primary contributions of this essay are as follows:

1. The ALSTM-FCN model was constructed to classify the common polluting gases, and the model parameters were optimized by SSA algorithm to improve the performance of the model.
2. Experiments demonstrate the importance of using SSA algorithm to optimize model parameters, and the performance of the model is evaluated on UCI datasets.
3. The accuracy, precision, recall rate, F1 score, and computation cost of the ALSTM-FCN model are thoroughly examined, and the model’s efficacy is further confirmed by contrasting it with the LSTM, FCN, DT, and other traditional models.

The organizational structure of this paper is as follows. The second part introduces the literature review of the algorithm model in electronic nose system. The third part introduces the network mode and dataset. The fourth part shows the evaluation indexes and experimental results of the algorithm model. In the fifth part, the experimental results are discussed. Finally, the sixth part summarizes and introduces the future work direction.

## Literature review

The electronic nose consists of a pattern recognition algorithm and a sensor array. Using multiple sensor arrays is more effective than using a single sensor to identify gas [12]. There are several different kinds of gas sensors, the most often used being metal oxide semiconductor (MOS) sensors [13]. The electronic nose’s identification performance may be effectively enhanced by using an appropriate pattern recognition algorithm. Popular algorithms for pattern recognition include PCA, LDA, SVM, and CA [14–17]. Machine learning algorithms are capable of achieving good results on tiny dataset because of their flexibility. Some of these are still widely used today because of their accessibility, usability, and low cost. However, the data that gas sensor arrays gather are often high-dimensional data, and it is challenging for the conventional pattern recognition algorithm to identify the intricate nonlinear connection between the data. As a consequence, processing big dataset is not sufficient. The field of deep learning has advanced quickly in recent years, artificial neural network (ANN) provides an opportunity for electronic nose to process high-dimensional sensor data, and its performance is significantly higher than that of traditional pattern recognition algorithms [18,19]. Recently, researchers have combined deep learning algorithms with sensor arrays and achieved good gas detection results. Lin et al. used lightweight residual CNN to identify soybeans with different origins with success [20]. Gamboa J et al. used CNN and SVM to identify target gasses [21]. Cai et al. used CNN-LSTM-AM model to carry out on-line detection of multi-gas in mud logging process [22]. To categorize liquor gas information, Hou et al. suggested a method for identifying binary codes using triangle differences [23].

The number of hyperparameters in a network model increases along with its complexity, thus selecting the right ones is essential to improving performance. Grid search algorithm is a common method to set model parameters, but it can be very time-consuming if there are many parameters. Therefore, scholars have proposed some other optimization algorithms, such as genetic (GA) algorithms, particle swarm optimization (PSO) algorithms, grey wolf optimization (GWO) algorithm, etc [24]. Among them, In the later stages, the convergence speed of PSO and GWO is sluggish, and it is easy to slip into the local optimum, and the efficiency of GA is low. The sparrow search algorithm that Xue and Shen suggested in 2020 is the one used in this study [25]. Compared with other optimization algorithms, because of its powerful optimization capabilities and quick convergence time, SSA has garnered a lot of interest. The distributed generations optimal configuration model was solved by Wang et al. using SSA, and the effectiveness and superiority of SSA were validated by experimental simulation [26].

Key information to increase classification performance may be found in the depth information that a neural network learns from sensor data. However, it also contains extraneous data to worsen classification accuracy. To solve this problem, researchers have found that combining attention mechanisms with neural networks can effectively enhance the network model’s ability to learn important information. Currently, image recognition and other domains make extensive use of the attention mechanism [27]. Among the primary attention methods are squeeze-and-excitation (SE) [28], efficient channel attention (ECA) [29], convolutional block attention module (CBAM) [30], coordinate attention (CA) [31], etc. In recent years, many researches were carried out that combined attention mechanism with neural network. Yan et al. classified rice gas information using a channel-space cooperative attention technique [11]. Zhang and colleagues classified gas information of various spirits using a channel-space cooperative attention approach [32]. Dynamic attention processes were used by Men et al. to determine the rice quality at various storage times [33].

## Methods

The dataset utilized for the simulation tests in this work came from the UCI. This dataset contains a variety of hazardous gases commonly found in industrial production. Each and every algorithm was developed using the Python 3.6 integrated development environment (PyCharm 2023.1.1, Community Edition) to guarantee consistency in the training and testing of the models. All programs were performed on Windows 10 (x64) operating system (GPU: NVIDIA GTX 1080Ti, RAM: 32G). GPU parallel compute is used to accelerate the deep learning training process. TensorFlow was used as the primary computational tool. Here, the SSA, LSTM, FCN and attention mechanism are first reviewed.

### Sparrow search algorithm (SSA)

A novel population intelligence optimization technique called the Sparrow search algorithm was put out by XUE J in 2020 [25]. This method offers the benefits of great optimization ability and rapid convergence speed over other optimization algorithms. The anti-predation and foraging strategies of sparrows serve as an inspiration for SSA. Typically, sparrows are split into producers and followers in order to finish the search. Since producers have greater resources, it is their responsibility to identify areas that are abundant in food and provide instruction to followers. When the producer spots an opponent, he directs his people in other directions. While producers and followers undergo identity changes during iterations, the overall proportion of producers to followers does not vary.The producer’s location has been revised as follows:

$$X_{i,j}^{t+1}=\{\begin{array}{ll} X_{i,j}^{t}\cdot \exp(\frac{-i}{\alpha \cdot iter_{\max}}) & {\mathrm{if}\;R}_{2}<\mathrm{SN}\\ X_{i,j}^{t}+L\cdot K & {\mathrm{if}\;R}_{2}\geq SN \end{array}$$ (1)

The maximum number of repetitions is denoted by K, and the random integer L has a typical normal distribution. In the interval of (0, 1], α is a uniformly random number. K indicates a matrix of 1*d, with each element being 1. The ith sparrow’s location in the jth dimension during the tth iteration is indicated by $X_{i,j}^{t+1}$. A population of sparrows has faced danger and has to take action if the warning value (R_2_) is achieved (R_2_∈ [0, 1]). When the number of sparrows is within the safety limit (SN ∈ [0.5, 1]), it may travel properly. This is known as the safety value.

Scrounger Location Updates:

$$X_{i,j}^{t+1}=\{\begin{array}{ll} L\cdot \exp(\frac{X_{wost}^{t}‐X_{i,j}^{t}}{i^{2}}) & if\;i>n/2\\ X_{P}^{t+1}+|X_{i,j}^{t}-X_{P}^{t+1}|\cdot A^{+}\cdot M & otherwise \end{array}$$ (2)

The best producer at iteration t+1 is indicated by the letter $X_{P}^{t+1}$. $X_{worst}^{t}$ represents the worst position at the tth iteration; L is a random integer that follows the conventional normal distribution; and M stands for a 1*d matrix with 1 as each entry. A is a 1*d matrix where each element has a random assignment of either 1 or −1.

Location updates for early-warning agents:

$$X_{i,j}^{t+1}=\{\begin{array}{ll} X_{best}^{t}+\beta \cdot |X_{i,j}^{t}-X_{best}^{t}| & if\;f_{i}>f_{g}\\ X_{i,j}^{t}+P\cdot (\frac{X_{i,j}^{t}-X_{worst}^{t}}{(f_{i}-f_{w})+\varepsilon }) & if\;f_{i}=f_{g} \end{array}$$ (3)

$X_{best}^{t}$ represents the present global ideal location, and β is a random variable that follows a normal distribution. The movement path and step control parameters of the sparrow are indicated by the letter P, and P ∈ (−1, 1). The current adaption value for a single sparrow is *f_i_*. At now, *f_w_* represents the worldwide worst adaptation value, while *f_g_* represents the global ideal value. The tiny constant ε keeps the denominator from becoming zero.

### Long short-term memory recurrent neural network (LSTM-RNN)

**Fig 1:**
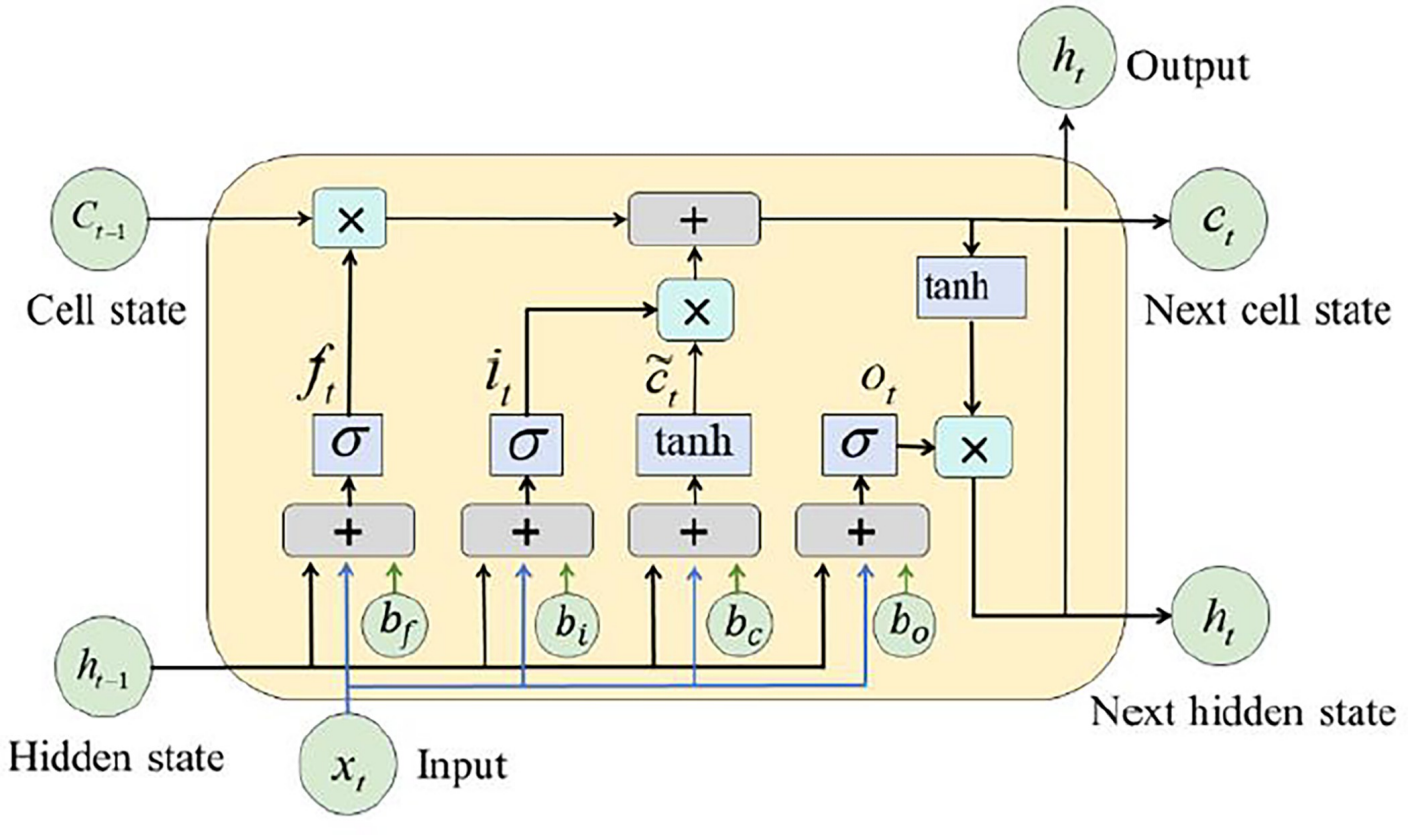
Architecture of a LSTM.

The LSTM neural network, a kind of recurrent neural network (RNN), was first presented by Hochreiter and Schmidhuber in 1997 [34]. Unlike feedforward neural networks, long-term input analysis is possible with LSTM. Using LSTM, information in a lengthy time series may be effectively described and transmitted without losing track of crucial historical information. Concurrently, LSTM can tackle the gradient disappearance/explosion issue of RNN. Fig 1 illustrates the forgetting gate, input gate, and output gate that comprise the memory cell unit and allow for selective information flow [35]. While maintaining important information in the memory cell state, the forget gate has the ability to selectively forget certain information. Incoming data flow management and selective memory cell state data storage are two of the input gate’s duties. All data produced from the memory cell is guaranteed to be in sync with the current time by the output gate.These three gate designs perform memory cell operations, such matrix multiplication and nonlinear summation, to keep memory from being lost during computation iterations.

Typically, the LSTM unit performs the following computations:

$$i_{t}=\sigma (G_{i}x_{t}+L_{i}h_{t-1}+b_{i})$$ (4)

$$o_{t}=\sigma (G_{o}x_{t}+L_{o}h_{t-1}+b_{o})$$ (5)

$$f_{t}=\sigma (G_{f}x_{t}+L_{f}h_{t-1}+b_{f})$$ (6)

$${\tilde{c}}_{t}=\tanh(G_{c}x_{c}+L_{c}h_{t-1}+b_{c})$$ (7)

The weights of the input data for each gate are represented by *G_i_*, *G_o_*, *G_f_* and *G_c_*. The preceding state’s common recurrent weights are represented by *L_i_*, *L_o_*, *L_f_* and *L_c_*.The bias term is denoted by *b_i_*, *b_o_*, *b_f_* and *b_c_*, while the sigmoid activation function is expressed as *σ*. The following formula is used to determine the state to be preserved at the current time step. Lastly, the concealed state that exists right now is displayed as follows:

$$c_{t}=f_{t}*c_{t-1}+i_{t}*{\tilde{c}}_{t}$$ (8)

$$h_{t}=\tanh(c_{t})*o_{t}$$ (9)

### Fully convolutional network (FCN)

CNN has shown its efficacy in addressing the temporal classification issue [36]. Upon input of the data, the convolutional kernel performs a sliding convolution at a designated stride length in order to extract the characteristics of the data. A CNN variation called FCN is able to address CNN’s overfitting and gradient explosion issues. FCN employs a global average pool layer (GAP) in place of the conventional final full connection (FC) layer and switches from CNN’s full connection layer to the convolutional layer without a local pooling layer [37]. Convolutional layers are the fundamental building blocks of FCN, and each one is capable of applying nonlinear changes to the input time series. After the convolutional layer, the gathered features are routed to the global average pooling layer, which is followed by the batch normalization (BN) layer and the rectifying linear unit (ReLU) activation layer. The softmax layer, which creates category tags, is the last layer to be linked.

### Attention mechanism

As a result of its little memory, LSTM’s performance may quickly decline when handling long-term dependencies in a lengthy sequence. This problem can be solved through the attention mechanism, the concept of attention proposed by Bahdanau et al [38]. The attention mechanism is inspired by the visual system of animals, which allows animals to focus on specific observation objects. Applying the attention mechanism to the LSTM can improve the robustness of the model by making the network focus on those features that are relevant to the output and ignore those that interfere with the information. The output element *c_i_* is determined by a input sequence (*u_1_*, *u_2_*, …, *u_n_*), where n represents the maximum length of the input sequence. Each annotation *u_i_* contains information for the entire input sequence, focusing on the elements around the *i_th_* element in the input sequence, *c_i_* can be represented by the Formula 10.


$$c_{i}=\overset{n}{\underset{j=1}{\sum }}G_{ij}u_{j}$$ (10)

Where, *G_ij_* represents the weight of each annotations *u_i_*, and the weight is calculated as follows:

$$G_{ij}=\frac{\exp(u_{i}^{T}u_{j})}{\sum_{k=1}^{n}\exp(u_{i}^{T}u_{k})}$$ (11)

*u_i_* and *u_j_* represent the *i_th_* and *j_th_* annotations, and T represents the transpose of the vector.

### ALSTM-FCN

**Fig 2:**
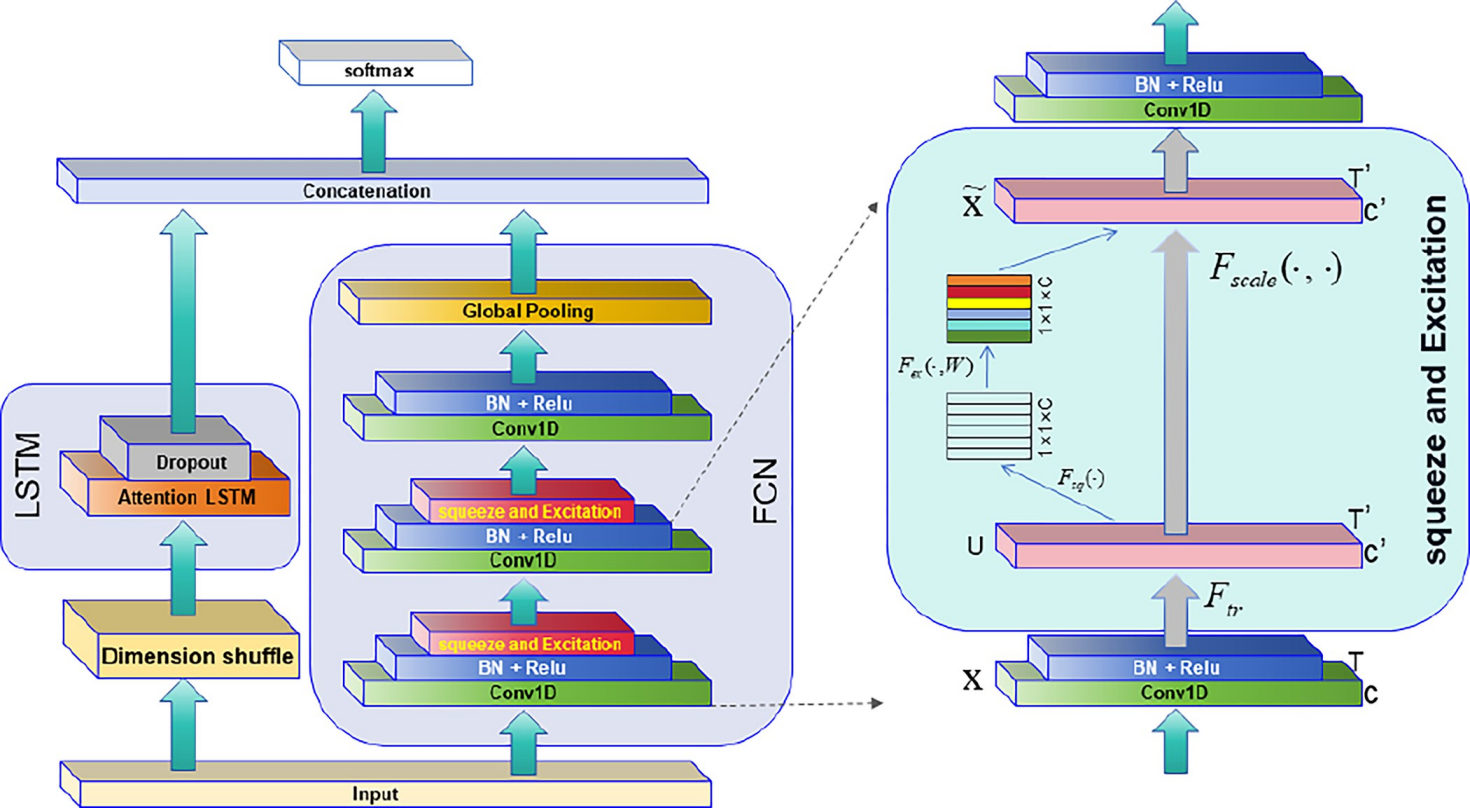
The ALSTM-FCN framework.

In the proposed attention-based LSTM-FCN model, the input data is routed across two parallel networks: LSTM and FCN. The output from the two networks is concatenated and categorized using a softmax layer. The gas classification flow chart based on ALSTM-FCN is shown in Fig 2.

LSTM and FCN adopt parallel structure to extract features from data respectively, and finally merge them into one vector. In addition, The FCN contains three time convolution modules, and the convolution block contains one convolution layer, which has multiple filters (128,256,128), and the kernel sizes are 8,5,3. Each convolution layer is succeeded by batch normalization, and the batch normalization layer is succeeded by the ReLU activation function.The squeeze and excitation is introduced behind every two convolution blocks. Extrusion and excitation module is proposed by Hu et al [28]. This method measures the importance of each feature channel and inhibits those features with less influence, thus improving the classification effect of the model. Fig 2 provides an overview of the steps involved in computing the squeeze-and-excite block in our design. The construction and operation of the suggested model on the Python platform are shown in Table 1.

**Table 1:** The ALSTM-FCN layer’s structure.

Layer (Type)	Output Shape	Parameters	Connected to
input_1 (Input Layer)	(None, 1, 24)	0	
permute_1 (Permute)	(None, 24, 1)	0	input_1[0][0]
conv1d_1 (Conv1D)	(None, 24, 128)	1152	permute_1[0][0]
batch_normalization_1	(None, 24, 128)	512	conv1d_1[0][0]
activation_1 (Activation)	(None, 24, 128)	0	batch_normalization_1[0][0]
global_average_pooling1d_1	(None, 128)	0	activation_1[0][0]
reshape_1 (Reshape)	(None, 1, 128)	0	global_average_pooling1d_1[0][0]
dense_1 (Dense)	(None, 1, 8)	1024	reshape_1[0][0]
dense_2 (Dense)	(None, 1, 128)	1024	dense_1[0][0]
multiply_1 (Multiply)	(None, 24, 128)	0	activation_1[0][0]
			dense_2[0][0]
conv1d_2 (Conv1D)	(None, 24, 256)	164096	multiply_1[0][0]
batch_normalization_2	(None, 24, 256)	1024	conv1d_2[0][0]
activation_2 (Activation)	(None, 24, 256)	0	batch_normalization_2[0][0]
global_average_pooling1d_2	(None, 256)	0	activation_2[0][0]
reshape_2 (Reshape)	(None, 1, 256)	0	global_average_pooling1d_2[0][0]
dense_3 (Dense)	(None, 1, 16)	4096	reshape_2[0][0]
dense_4 (Dense)	(None, 1, 256)	4096	dense_3[0][0]
multiply_2 (Multiply)	(None, 24, 256)	0	activation_2[0][0]
			dense_4[0][0]
conv1d_3 (Conv1D)	(None, 24, 128)	98432	multiply_2[0][0]
batch_normalization_3	(None, 24, 128)	512	conv1d_3[0][0]
attention_lstm_1	(None, 128)	110336	input_1[0][0]
activation_3 (Activation)	(None, 24, 128)	0	batch_normalization_3[0][0]
dropout_1 (Dropout)	(None, 128)	0	attention_lstm_1[0][0]
global_average_pooling1d_3	(None, 128)	0	activation_3[0][0]
concatenate_1 (Concatenate)	(None, 256)	0	droout_1[0][0]
			global_average_pooling1d_3[0][0]
dense_5 (Dense)	(None, 6)	1542	concatenate_1[0][0]

### Parameter optimization

**Fig 3:**
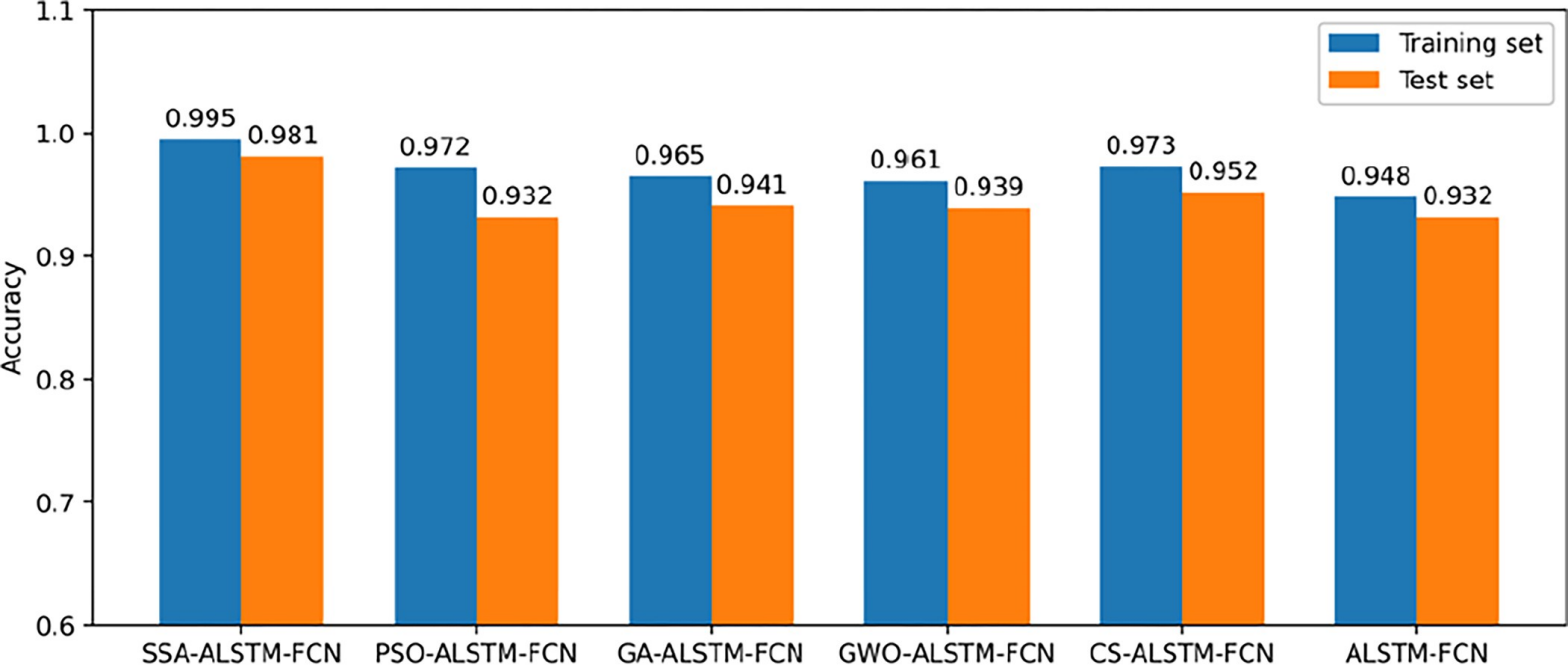
Classification accuracy of different optimization algorithms and prediction models.

In machine learning and deep learning, hyperparameter selection is an important part of model training. Choosing an optimization strategy that automatically adjusts hyperparameters is crucial if you want to increase the model’s classification accuracy. Hyperparameters refer to parameters that need to be determined before the model is trained, such as the learning rate, the maximum depth of the decision tree, the number of layers of the neural network, etc. In this study, SSA is selected to optimize the Learning rate, Dropout and Batch size of the model. The parameters of SSA are as follows: sparrow population is 50, the maximum number of iterations is 40, the proportion of discoverers is 0.2, the proportion of watchers is 0.1, alarm value is 0.8, popmax = 5, popmin = −5. The range of the SSA to search the learning rate parameter is [0.0001, 0.01], the range of the search Dropout is [0.1,1], and the range of the search Batch size is [10,200]. The optimized parameters are as follows: the learning rate is 0.0005, the value of Dropout is 0.8 and the value of Batch size is 128. In order to further verify the rationality of SSA algorithm, the performance improvement effects of a single ALSTM-FCN model, particle swarm algorithm, and five optimization algorithms on ALSTM-FCN models (PSO, GA, GWO, CS, and SSA) were compared separately. To guarantee the generalization performance and rationality of model Classification results, all models are averaged from the results of 10 runs. Fig 3 shows the classification results optimized by other four optimization algorithms.

It can be found that the accuracy rate of the ALSTM-FCN model optimized by algorithm is higher than that of a single ALSTM-FCN model. There are slight differences between the results of the training set and the test set of the SSA-ALSTM-FCN model, and the optimization of the ALSTM-FCN model has a better degree of generalization than other algorithms, so SSA is more suitable for establishing accurate gas classification models.

## Results

### Data preparation

The data collection "Gas Sensor Array Drift Dataset at Different Concentrations" from the UCI is used in this work, the access link is http://archive.ics.uci.edu/ml/datasets/Gas+Sensor+Array+Drift+Dataset+at+Different+Concentrations.The dataset includes four distinct kinds of gas sensors: TGS-2600, TGS-2602, TGS-2610, and TGS-2620 and there are four sensors of each kind. These gas sensors have different sensitivity levels based on the kind of gas. Table 2 lists the selectivity of gas sensors.

**Table 2:** Overview of the gas sensors.

ID	Type	Main Detect Gas
1	TGS2600	hydrogen monoxide, carbon monoxide, air pollutants
2	TGS2602	ammonia, hydrogen sulfide, toluene, air pollutants
3	TGS2610	propane, butane, alcohol, air pollutants
4	TGS2620	alcohol, solvent vapors, air pollutants

The sensor array was housed in a sealed 60 mL box for the experiment. A steady 200 mL/min flow rate was used to inject the gas sample. At a sample frequency of 100 Hz, the conductivity (S/m) of the sensor is continually gathered. Every measurement is created by continually collecting data from a set of sixteen sensors. The gas sample contains six different concentrations of gases: ammonia, acetaldehyde, acetone, ethylene, ethanol and toluene, corresponding to the concentration intervals of (50,1000), (5,500), (12,1000), (10,300), (10,600) and (10,100) ppm. The dataset contains 13910 gas samples, each with a length of 128.

**Fig 4:**
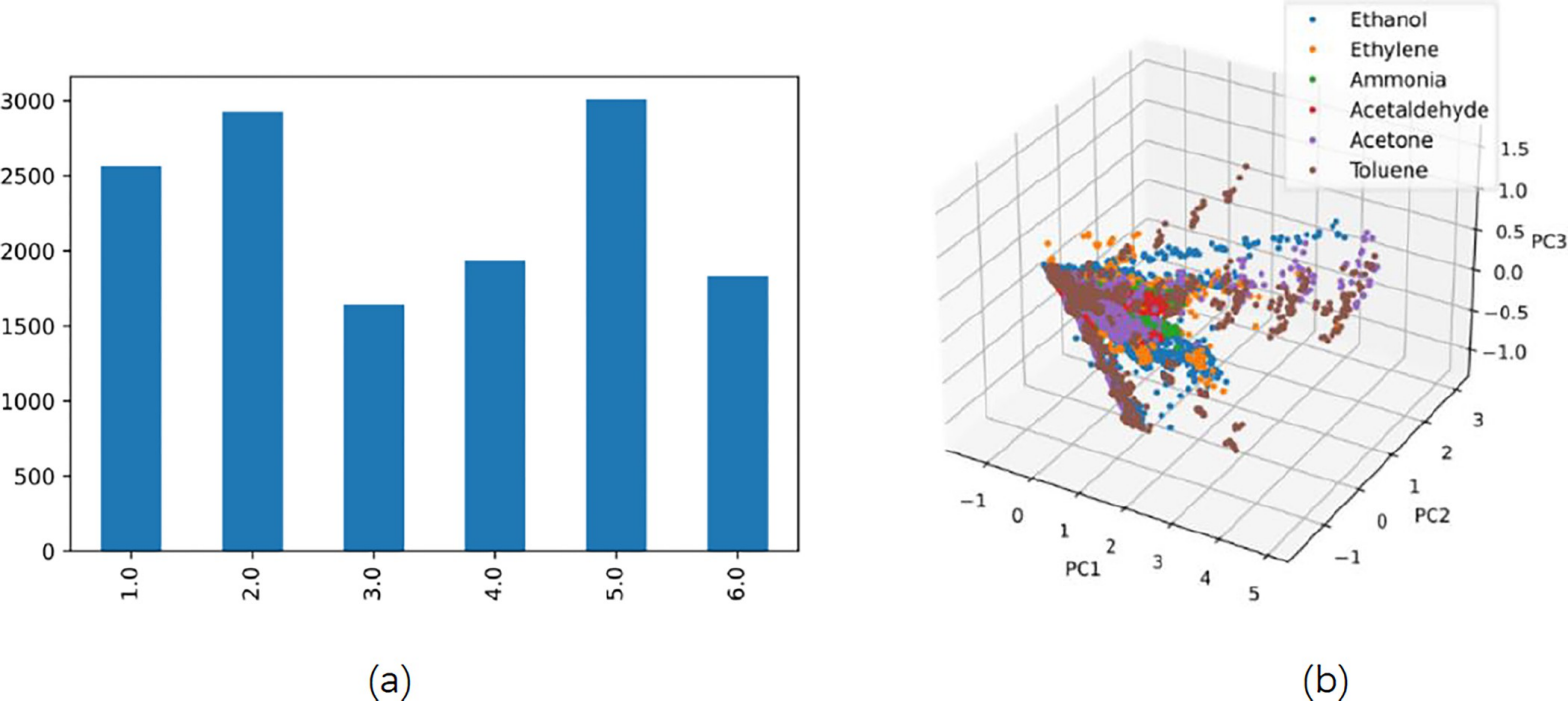
(a) The number of six gas samples in the dataset; (b) Data distribution of gas samples.

For convenience, the six gases are numbered from 1 to 6. The specific numbering information and model classification label are shown in Table 3. A common method for visualizing multi-dimensional data from gas sensor arrays is principal component analysis (PCA) [39], the quantity and distribution of gas samples in the dataset are shown in Fig 4, and more details about the dataset can be found in [40].

**Table 3:** The label of six gases.

Gas class	number	Classification Labels of model
ethanol	1	000001
ethylene	2	000010
ammonia	3	000100
acetaldehyde	4	001000
acetone	5	010000
toluene	6	100000

### The Whole experimental process

**Fig 5:**
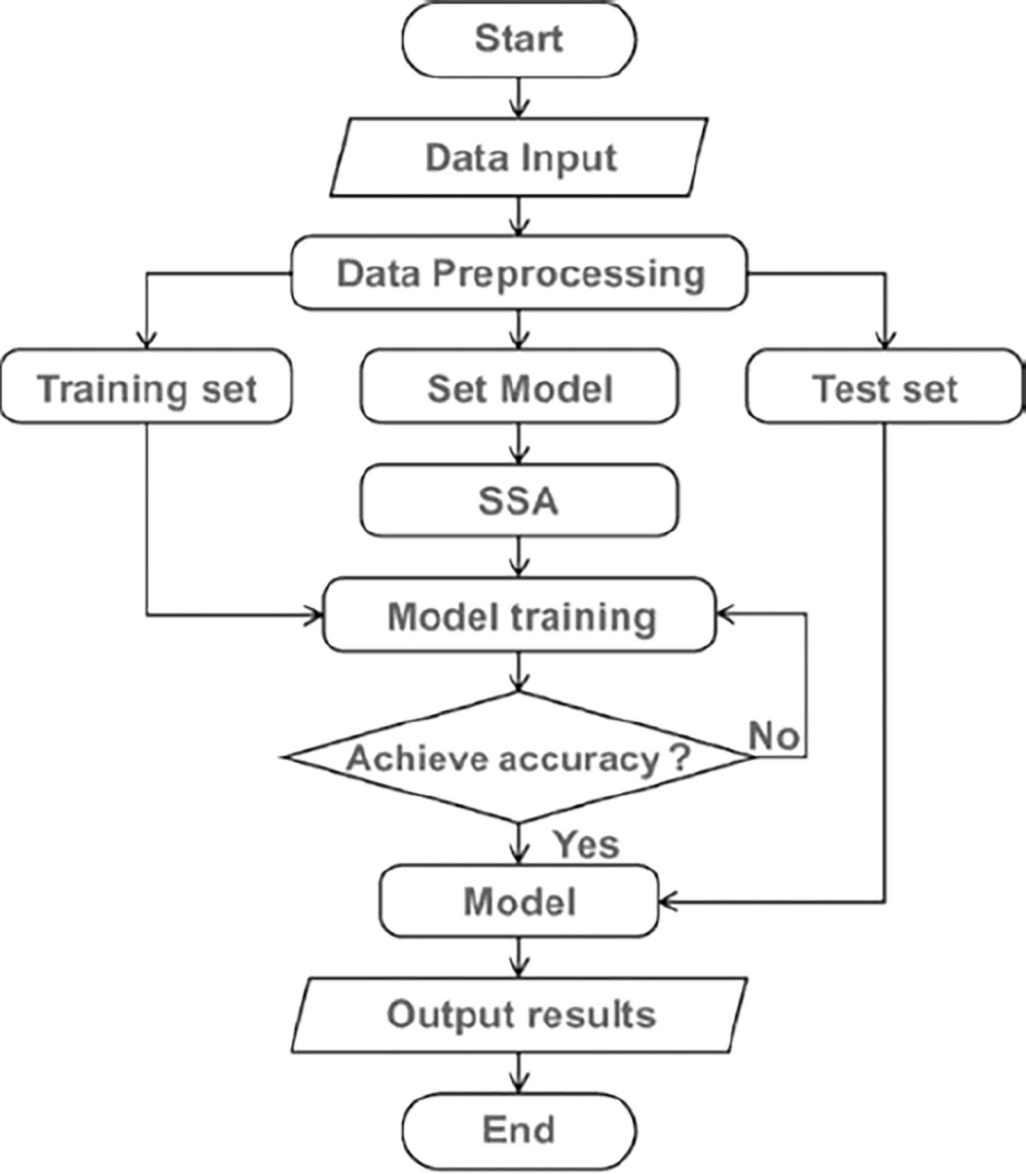
The flow chart of the whole experiment.

The experiment described in this article was carried out in the following manner: The data is first preprocessed and analyzed, and then it is split into training and test sets. Next, the ALSTM-FCN model is created, and the processed data is fed into the model to be trained. During the training phase, the model’s parameters are optimized using the SSA. Finally, the test set is used to evaluate the trained model’s performance. Fig 5 shows the whole experimental flow chart.

### Data processing

Because sensor response sizes vary widely, normalization helps prepare appropriate data for the classification model’s input space and prevent prediction mistakes caused by significant discrepancies across sensor outputs. Within the experiment, the data is normalized to a range of 0 to 1, which increases the classification model’s speed and accuracy.


$$y_{i}=\frac{x_{i}-x_{\min}}{x_{\max}-x_{\min}}$$ (12)

Here, *x_i_* represents the initial data, the response data’s lowest value is represented by *x_min_*, while its highest value is represented by *x_max_*.

**Fig 6:**
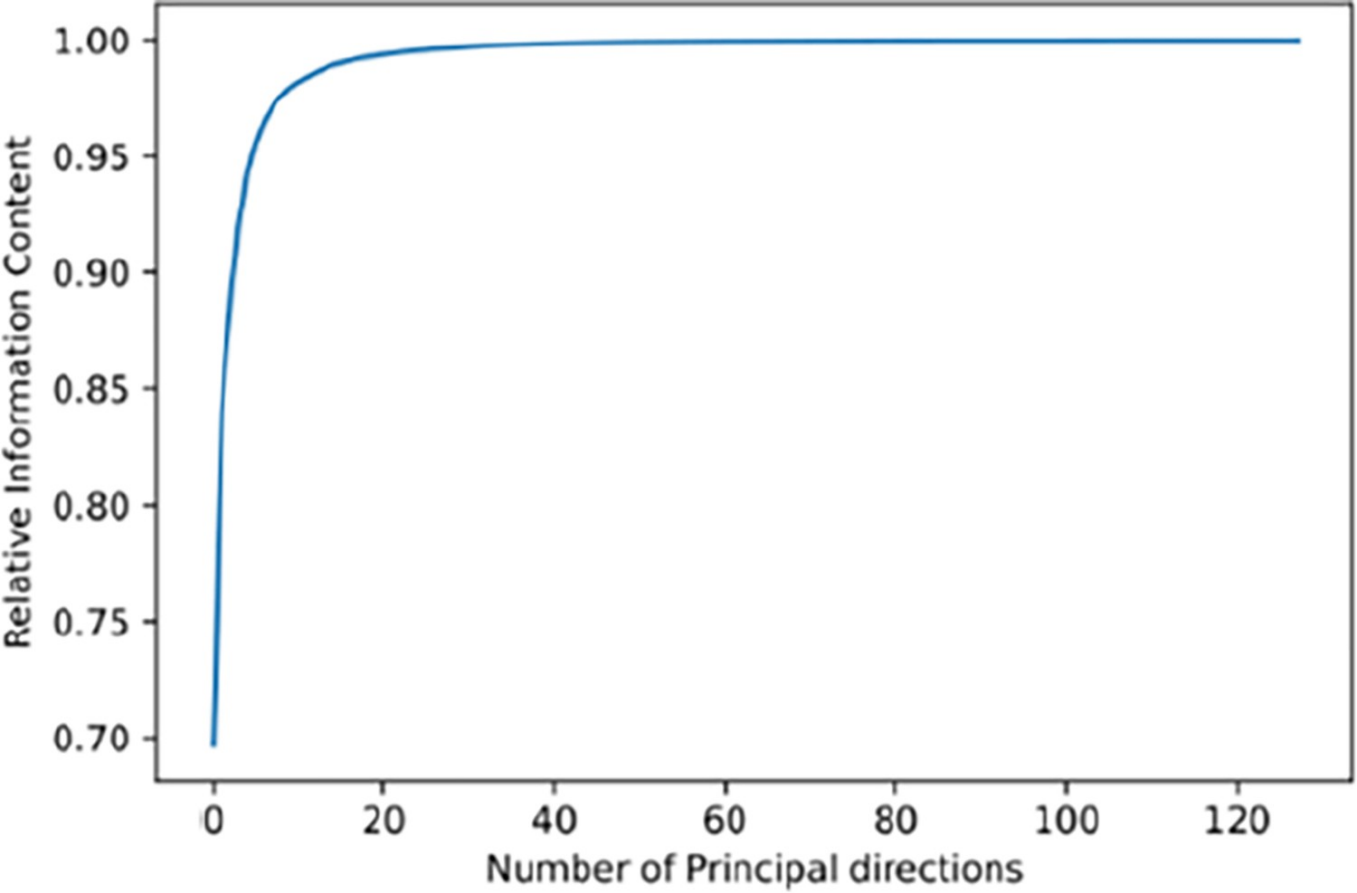
Variation of RIC with number of chosen principal directions.

Using singular value decomposition to break down a matrix into a collection of uncorrelated variables known as principle components, PCA is a popular technique for processing high-dimensional feature data. It eliminates noise and unwanted characteristics from the data while keeping the most significant features, thereby improving the speed of data processing [41]. As expected, in the first few major directions, the Relative Information Content (RIC) value rapidly approaches its maximum possible value of 1.00, as shown in Fig 6.

Actually, 99.5% of the RIC value may be produced using only the first 24 major directions. In order to create the low-dimensional space, the basis vectors for the first 24 directions’ unit vectors are chosen. Currently, a 13910 * 24-dimensional feature matrix has been created by integrating the data. Following the reduction of the data using PCA, the samples were split into training and test sets in an 8:2 ratio.

### Gas classification identification

**Fig 7:**
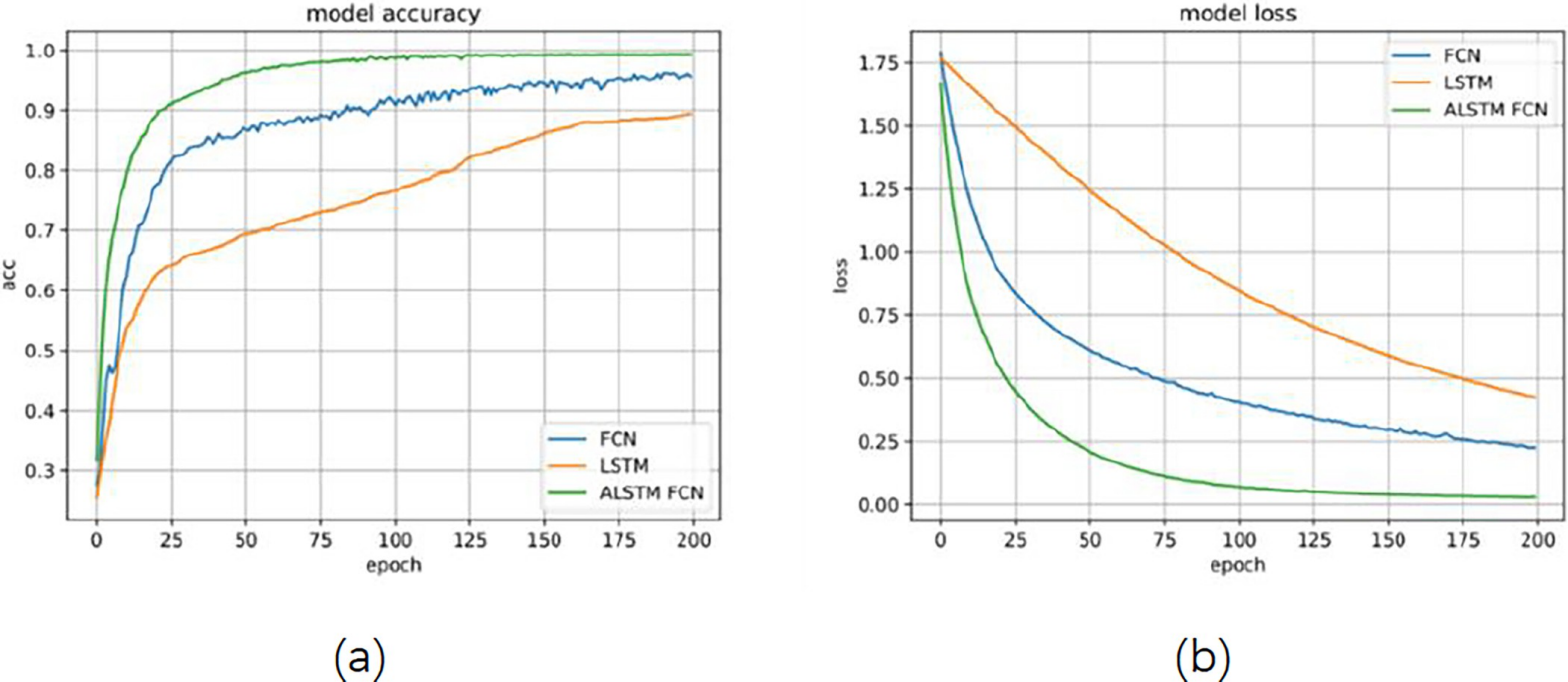
Gas classification results of three models: (a) the accuracy curve; (b) the loss curve.

**Fig 8:**
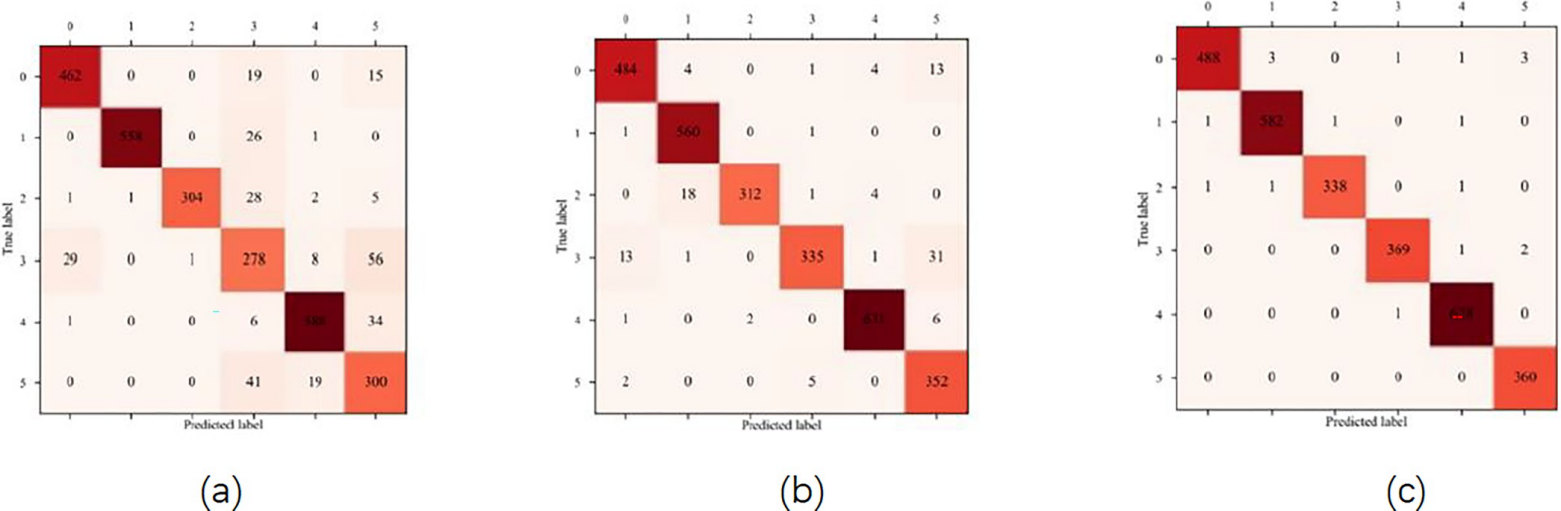
(a) the confusion matrix of LSTM; (b) the confusion matrix of FCN; (c) the confusion matrix of ALSTM-FCN.

We initially contrasted ALSTM-FCN with the LSTM and FCN models in order to assess the viability of the suggested approach. Learning curve is an important index algorithm for evaluating deep learning. As shown in Figs 7 and 8, we created accuracy curves, loss curves, and confusion matrices for the three techniques. Compared to individual classifiers, the fused model presents the best.

**Fig 9:**
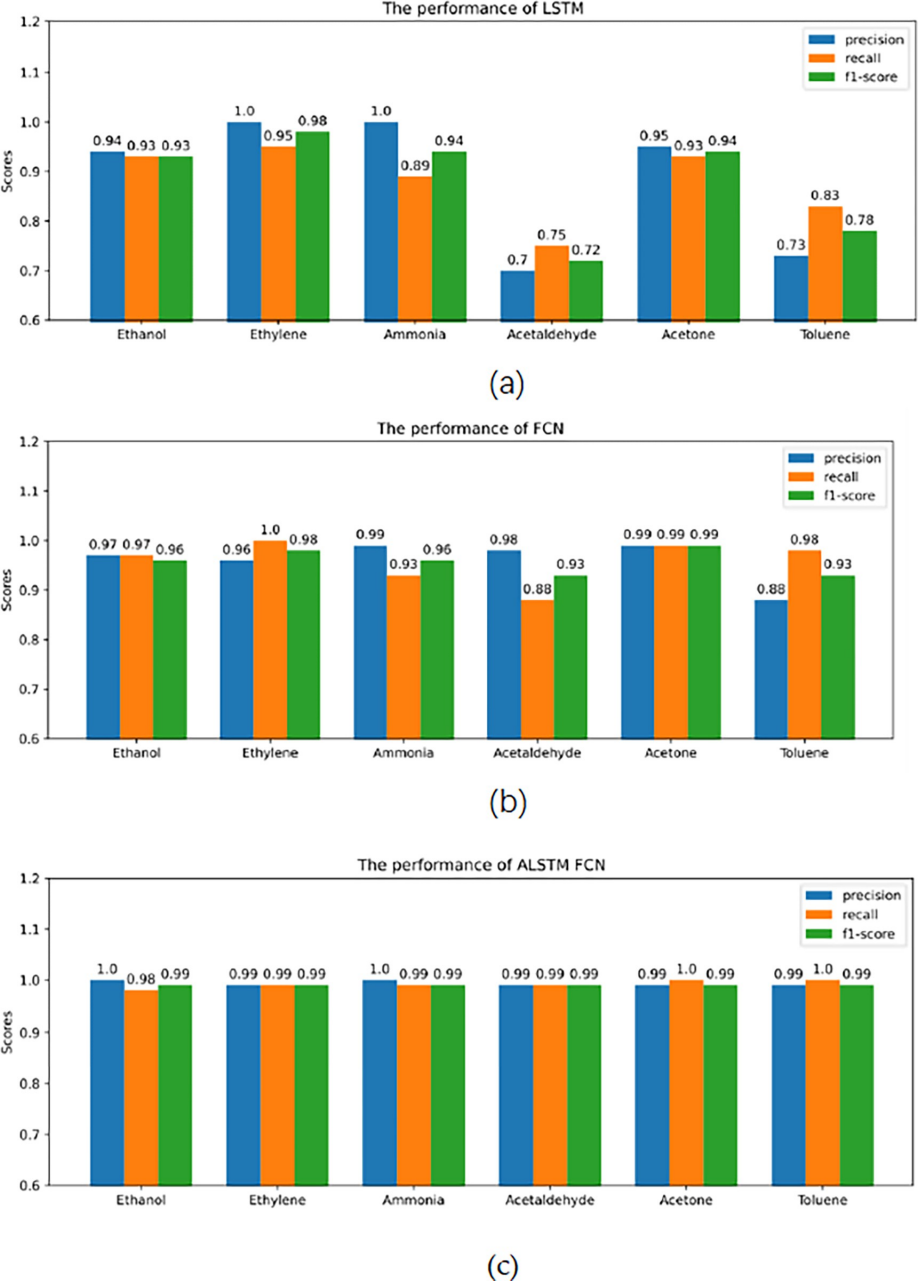
Gas classification results of three models: (a) the gas classification results of LSTM; (b) the gas classification results of FCN; (c) the gas classification results of ALSTM-FCN.


We evaluated the classification results of six gases using three indicators: precision (P), recall (R), and F1 score [42], in order to more accurately represent the three models’ capacity to recognize gases, as shown in Fig 9.

$$P=\frac{TP}{TP+FP}$$ (13)

$$R=\frac{TP}{TP+FN}$$ (14)

$$F1=2\times \frac{P\times R}{P+R}$$ (15)

Where, in that order, TP, FP, TN, and FN stand for true positive, false positive, true negative, and false negative, respectively.As shown in Fig 8, in three indices, the ALSTM-FCN model presented in this research outperforms the LSTM and FCN models, especially the classification effect of acetaldehyde is significantly improved.

We evaluated the performance of the ALSTM-FCN with a number of other models, including Ada Boost, K-nearest neighbor (KNN), random forest (RF), logistic regression (LR), extra tree (ET), and decision tree (DT), in order to further illustrate the efficacy of the model, all models were run 10 times, and the experimental results were shown in Table 4. We found that the proposed network has the highest accuracy of 99.461% and the lowest loss of 0.028, which is superior to other commonly used classification models.However, with the increase of network model complexity, the training time of ALSTM-FCN model is the longest (431 s), but all other indexes are better than other classification models, so this time consumption is acceptable.

**Table 4:** Comparison results of multi-models.

Classification model	Accuracy			Loss	Training time (s)
	Best	Mean	Std.		
Ada Boost	61.827	59.198	1.483	1.335	49
KNN	89.575	88.743	0.764	0.420	44
RF	98.454	97.478	0.518	0.152	61
LR	94.177	92.475	1.034	0.343	57
ET	98.167	97.046	0.729	0.255	46
DT	95.148	94.597	0.499	1.675	58
LSTM	89.471	88.417	0.423	0.425	203
FCN	96.083	95.799	0.328	0.226	362
ALSTM-FCN	99.461	98.148	0.292	0.028	431

## Discussion

Electronic nose system has been widely used in the field of environmental engineering [43], and different algorithm models will have a great impact on the performance of electronic nose. In this study, the application of ALSTM-FCN model in electronic nose system is proposed for the first time to accurately classify a variety of common harmful gases. Previous studies have proved that using optimization algorithms to optimize the parameters of the model can significantly improve the performance of the model. For example, Sharma et al. used the CNN model optimized by GWO algorithm to detect sugarcane diseases [44], Zhang et al. used PSO algorithm to optimize SVM and successfully distinguished wheat grades [45]. In this study, the SSA algorithm proposed in recent years was also used to optimize the hyperparameters of the model, as shown in Fig 3. After optimizing the parameters of the model by using optimization algorithms, the classification accuracy can indeed be improved. In addition, SSA can effectively solve problems trapped in local optimal solutions, which is superior to classical optimization algorithms such as PSO and GWO.

Previous studies often used a single model for data processing, for example, Zhang et al. used the LSTM model for gas leakage detection [46], Shin et al. used the FCN model to monitor indoor air quality [47]. In contrast, the fusion model proposed in this study has obvious advantages in extracting important features of data. As shown in Fig 7, the ALSTM-FCN model has the best classification performance, and the convergence speed is higher than that of the single model (LSTM and FCN). It may be because FCN makes up for LSTM’s lack of learning ability for high-dimensional features.

In addition, when the gas sensor performs gas acquisition for a long time, the baseline drift phenomenon will occur [48]. In previous studies, Se et al. constructed a sensor drift compensation framework to realize multi-tasking of sensor drift and gas classification [49], Oh et al. added a drift compensation module to the classification model to improve the accuracy of gas classification [50]. While the ALSTM-FCN model proposed in this study can accurately capture the characteristics of various gas data without compensating the drift of the dataset, and the gas classification result reaches 99.461%. Compared with the classical classification algorithms such as DT and RF, in addition to the longer training time of the model, the other indexes get the best results.

## Conclusions and future works

In this paper, a multi-gas classification model of LSTM-FCN based on attention mechanism is proposed, which is used to detect and classify many common toxic and harmful gases in industrial production.The model models time dependency using LSTM and extracts advanced features using FCN. By combining the attention mechanism with the model, important information can be captured and given higher weights, thus improving classification accuracy. In order to further improve the performance of the model, we compared SSA, PSO, GWO and other optimization algorithms, and the results show that SSA algorithm has better generalization. We trained and tested the model on UCI datasets, after SSA optimization, the average recognition accuracy of the model for all gases reached 99.461%. We compared ALSTM-FCN model with LSTM, FCN, KNN and other classical classification models, and the results show that ALSTM-FCN model has the highest average accuracy (98.148%). This work has some potential application value and offers valuable recommendations for the sensor array-based detection of hazardous Agasses. Although the ALSTM-FCN model proposed in this paper for a variety of toxic gases can achieve a good classification effect, due to the complexity of the proposed model, the network parameters are more than those of classical machine learning models, so there is still room for improvement in training time cost.

In our future work, We will further improve the data preprocessing method through more extensive experiments to improve the time efficiency of training the model. In addition, we hope that the proposed method can be applied to different applications and different gas sensor systems to detect more harmful gases in production and life, so as to further evaluate their classification performance.

## Supporting information

S1 File A file containing all the experimental data. (DOCX)
