# Supplementary material for: Multi-gas pollutant detection based on sparrow search algorithm optimized ALSTM-FCN
Source: PLoS One. 2024 Sep 13;19(9):e0310101. doi: 10.1371/journal.pone.0310101 (PMC11398686; doi:10.1371/journal.pone.0310101)
Supplement: S1 File — (DOCX) [file pone.0310101.s001.docx]

1. Te classification accuracy of different optimization algorithms and prediction models.

| **Algorithm** | **SSA** | **PSO** | **GA** | **GWO** | **CS** |
| --- | --- | --- | --- | --- | --- |
| Training set | 0.995 | 0.972 | 0.965 | 0.961 | 0.973 |
| Test set | 0.981 | 0.932 | 0.941 | 0.939 | 0.952 |

1. The results of gas classification by LSTM, FCN and ALSTM-FCN

**The gas classification results of LSTM**

| **Gas** | **Ethanol** | **Ethylene** | **Ammonia** | **Acetaldehyde** | **Acetone** | **Toluene** |
| --- | --- | --- | --- | --- | --- | --- |
| precision | 0.94 | 1.00 | 1.00 | 0.70 | 0.95 | 0.73 |
| recall | 0.93 | 0.95 | 0.89 | 0.75 | 0.93 | 0.83 |
| f1-score | 0.93 | 0.98 | 0.94 | 0.72 | 0.94 | 0.78 |

**The gas classification results of FCN**

| **Gas** | **Ethanol** | **Ethylene** | **Ammonia** | **Acetaldehyde** | **Acetone** | **Toluene** |
| --- | --- | --- | --- | --- | --- | --- |
| precision | 0.97 | 0.96 | 0.99 | 0.98 | 0.99 | 0.88 |
| recall | 0.97 | 1.00 | 0.93 | 0.88 | 0.99 | 0.98 |
| f1-score | 0.96 | 0.98 | 0.96 | 0.93 | 0.99 | 0.93 |

**The gas classification results of ALSTM-FCN**

| **Gas** | **Ethanol** | **Ethylene** | **Ammonia** | **Acetaldehyde** | **Acetone** | **Toluene** |
| --- | --- | --- | --- | --- | --- | --- |
| precision | 1.00 | 0.99 | 1.00 | 0.99 | 0.99 | 0.99 |
| recall | 0.98 | 0.99 | 0.99 | 0.99 | 1.00 | 1.00 |
| f1-score | 0.99 | 0.99 | 0.99 | 0.99 | 0.99 | 0.99 |

1. The test results of each gas classification model.

| **Classification model** | **Accuracy** | | | **Loss** | **Training time (s)** |
| --- | --- | --- | --- | --- | --- |
|  | **Best** | **Mean** | **Std.** |  |  |
| Ada Boost | 61.827 | 59.198 | 1.483 | 1.335 | 49 |
| KNN | 89.575 | 88.743 | 0.764 | 0.420 | 44 |
| RF | 98.454 | 97.478 | 0.518 | 0.152 | 61 |
| LR | 94.177 | 92.475 | 1.034 | 0.343 | 57 |
| ET | 98.167 | 97.046 | 0.729 | 0.255 | 46 |
| DT | 95.148 | 94.597 | 0.499 | 1.675 | 58 |
| LSTM | 89.471 | 88.417 | 0.423 | 0.425 | 203 |
| FCN | 96.083 | 95.799 | 0.328 | 0.226 | 362 |
| ALSTM-FCN | 99.461 | 98.148 | 0.292 | 0.028 | 431 |

1. Access links to gas datasets used in this experiment.

<http://archive.ics.uci.edu/ml/datasets/Gas+Sensor+Array+Drift+Dataset+at+Different+Concentrations>.
